# Negative correlation between acetyl-CoA acyltransferase 2 and cetuximab resistance in colorectal cancer: Correlation between ACAA2 and cetuximab resistance in CRC

**DOI:** 10.3724/abbs.2023111

**Published:** 2023-06-13

**Authors:** Yitao Yuan, Xun Sun, Mengling Liu, Suyao Li, Yu Dong, Keshu Hu, Jiayu Zhang, Bei Xu, Sining Ma, Hesheng Jiang, Pengcong Hou, Yufu Lin, Lu Gan, Tianshu Liu

**Affiliations:** 1 Department of Medical Oncology Zhongshan Hospital Fudan University Shanghai 200032 China; 2 Department of Obstetrics and Gynecology Zhongshan Hospital Shanghai 200032 China; 3 Department of Surgery Southwest Healthcare Southern California Medical Education Consortium Temecula Valley Hospital Temecula USA; 4 Shanghai Institute of Precision Medicine Shanghai Ninth People’s Hospital Shanghai Jiao Tong University School of Medicine Shanghai 200032 China; 5 Department of Oncology Zhongshan Hospital (Xiamen) Fudan University Xiamen 361004 China; 6 Fudan Zhangjiang Institute Shanghai 200032 China; 7 Center of Evidence Based Medicine Fudan University Shanghai 200032 China

**Keywords:** colorectal cancer, cetuximab resistance, ACAA2, *Kras* mutation

## Abstract

The emergence of anti-EGFR therapy has revolutionized the treatment of colorectal cancer (CRC). However, not all patients respond consistently well. Therefore, it is imperative to conduct further research to identify the molecular mechanisms underlying the development of cetuximab resistance in CRC. In this study, we find that the expressions of many metabolism-related genes are downregulated in cetuximab-resistant CRC cells compared to their sensitive counterparts. Specifically, acetyl-CoA acyltransferase 2 (ACAA2), a key enzyme in fatty acid metabolism, is downregulated during the development of cetuximab resistance. Silencing of *ACAA2* promotes proliferation and increases cetuximab tolerance in CRC cells, while overexpression of ACAA2 exerts the opposite effect. RTK-Kras signaling might contribute to the downregulation of ACAA2 expression in CRC, and ACAA2 predicts CRC prognosis in patients with *Kras* mutations. Collectively, our data suggest that modulating ACAA2 expression contributes to secondary cetuximab resistance in Kras wild-type CRC patients. ACAA2 expression is related to *Kras* mutation and demonstrates a prognostic role in CRC patients with *Kras* mutation. Thus, ACAA2 is a potential target in CRC with *Kras* mutation.

## Introduction

Colorectal cancer (CRC) is a major global health problem with high morbidity and mortality rates [1]. Given its insidious symptoms and signs, many CRC patients are diagnosed at advanced stages that are not ideal for surgery. Neoadjuvant chemoradiotherapy of advanced CRC decreases the risk of metastasis, but it may be ineffective in some patients and may even delay their surgery due to the adverse effects [2]. The prognosis of metastatic CRC patients remains poor, with a 5-year survival rate of less than 20% [3]. The development of anti-epidermal growth factor receptor (anti-EGFR) monoclonal antibody, cetuximab or panitumumab, is a milestone in metastatic colorectal cancer treatments. However, its effectiveness is limited to Ras wild-type metastatic CRC (mCRC) patients of the left colon [4]. Even in these patients, drug resistance often develops after 7 to 10 months of treatment. Therefore, there is a need to investigate the mechanisms behind cetuximab resistance and identify biomarkers that predict treatment response.

The development of anti-EGFR resistance is a complex process that involves multiple genes and factors due to the heterogeneity of tumour tissues. *Kras* is a frequently mutated oncogene in CRC, with a mutation frequency of approximately 40%, and up to 95% of mutations occur in exons G12 and G13 [5]. Previous studies have shown that initial *Ras* mutant cells, which are resistant but not detected, can continue to proliferate [ 6, 7] and secondary mutations of *Kras* genes are typically responsible for cetuximab resistance [8]. However, the biological mechanisms behind them have not yet been fully clarified.

Many studies suggested that rewired metabolism caused by *Kras* mutation contributes to tumour progression and cetuximab resistance. For example, the removal of methylglyoxal (MGO), a byproduct of glycolysis, reversed the resistance of mutant *Kras* to cetuximab [9]. Mutation in *Kras* could also increase glutamine utilization to meet cellular demands [10], and combining an inhibitor of glutamine metabolism with cetuximab was found to be a promising novel approach for overcoming acquired cetuximab resistance in CRC [11]. Additionally, metabolic responses measured by 18F-fluorodeoxyglucose positron emission tomography/CT (FDG-PET/CT) have been suggested as an indicator of later responses and even the final survival outcome of CRC patients treated with cetuximab [12]. In patient-derived CRC spheres, enhanced Warburg effects favour cetuximab resistance [7]. These findings underscore a metabolic adaptive model that ensures sufficient energy and crucial building blocks for the development of cetuximab resistance in colorectal cancer. Thus, investigating the metabolic vulnerabilities of tumours and elucidating the mechanisms underlying cetuximab resistance could open up new avenues for managing mCRC that is refractory to current treatments.

Acetyl-CoA acyltransferase 2 (ACAA2) is an enzyme that catalyzes the conversion of fatty acids into acetyl-CoA through mitochondrial beta-oxidation and serves as a bridge molecule between fatty acid degradation and the TCA cycle. Bioinformatics analyses have identified ACAA2 as a prognostic factor in lower-grade glioma with *IDH* mutation [13]. ACAA2 also participates in cell apoptosis and transcription [14]. Attenuation of ACAA2 reduces cell death in fibroblast MRC5 cells, which promotes liver cancer cell proliferation [ 15, 16]. However, its role as an important metabolism-related gene in tumour progression and cetuximab treatment of CRC remains unknown.

In the present study, our results indicated that higher ACAA2 expression was related to a better cetuximab response in Kras wild-type CRC patients. Its expression might be influenced by RTK-Kras signaling and serve as a favorable prognostic biomarker in CRC patients with *Kras* mutations.

## Materials and Methods

### Drug-resistant strain establishment and cell culture

The human colorectal cancer cell lines DLD1, HCT116, RKO, NCIH508 and HEK-293T were purchased from the Cell Bank of Chinese Academy of Sciences (Shanghai Institutes for Biological Sciences, Shanghai, China). To generate cetuximab-resistant cells, Kras wild-type CRC cells (NCIH508-CS) were treated with increasing concentrations of cetuximab, resulting in a resistant cell line (NCIH508-CR). All cells (RKO, HCT116, DLD1, and NCIH508-CS/CR) were cultured in RPMI-1640 medium (HyClone, Logan, USA) supplemented with 10% fetal bovine serum (FBS; Gibco, Paisley, UK), 1% penicillin, and 1% streptomycin (Gibco).

### RNA sequencing

Total cellular RNA was extracted with Trizol reagent (Invitrogen, Carlsbad, USA). The RNA integrity and library quality were assessed on the Agilent Bioanalyzer 2100 system (Agilent Technologies, Santa Clara, USA). Transcriptome sequencing was performed by Novogene Biotech Co (Beijing, China) on an Illumina HiSeq X-Ten platform (Illumina, San Diego, USA). Differentially expressed genes were defined with the criterion of fold change>2.0 at *P*<0.05 and selected using the “LIMMA” package. Gene Ontology (GO) and Kyoto Encyclopedia of Genes and Genomes (KEGG) pathway analyses were performed using the “clusterProfiler” package.

### Lentivirus-mediated gene knockdown

The ACAA2 interference plasmid vector used was pLKO.1-TRC-copGFP-F2A-PURO with the sequence 5′-CCGGCACACCTGGTTCACGAATTAACTCGAGTTAATTCGTGAACCAGGTGTGTTTTTG-3′. HEK-293T cells were cultured in T25 culture flasks, washed twice with PBS and then trypsinized until the cells were round. The day of seeding was considered day 1. On day 2, cells were transfected at a density of 5×10 ^5^ cells/mL, with 2 mL of cell suspension added to each well of a 6-well plate. The cell density was confirmed to be 70%‒80% before transfection. Transfection of HEK-293T cells in single wells of 6-well plates was performed as follows: target plasmid (1 μg); packaging plasmid I (psPAX2; 0.5 μg); and packaging plasmid II (pMD2G; 0.5 μg). After 8‒10 h, 4 mL of fresh culture solution was added, and transfection efficiency was observed after 24 h. After collecting the virus supernatant produced at 48–72 h, the virus solution was filtered through a 0.22-μm filter. Cells were infected with the viral suspension and screened with puromycin (2 μg/mL for 48 h, until the cells in the nontransfected group died) to obtain a cell line with stable *ACAA2* knockout.

### Cell proliferation assay

The proliferation of CRC cells was assessed by Cell Counting Kit 8 (CCK-8) assay and colony formation assay. For the CCK-8 assay, cells were seeded in 96-well plates and cultured for 5 days. Then CCK-8 reagent (Dojindo, Tokyo, Japan) was added and the optical density (OD) values were measured at 450 nm after 1 h of incubation. The readings were normalized against the values obtained on the 1st day. For the colony formation assay, cells were seeded in 6-well plates (500 cells/well) and cultured for 14 days. Colonies were fixed with 4% paraformaldehyde for 20 min, stained with 0.1% crystal violet for 30 min, and quantified using ImageJ software (National Institute of Health, Bethesda, USA).

### Inhibition assay

To assess the inhibitory effects of drugs, cells in the logarithmic growth phase were seeded in 96-well plates at a density of 4000 cells/well. The medium was replaced by medium containing different concentration gradient of drugs and treated for 72 h after the cells attached. The drug-containing medium was discarded and replaced by culture medium containing 10% CCK8. Finally, the absorbance was measured at 450 nm after 1 h of incubation.

### Quantitative real-time PCR analysis

After discarding the cell culture medium, the cells were washed 3 times with PBS, and 1 mL Trizol was added per dish. The cells were scraped, collected into EP tubes, and kept on ice for 10 min. After addition of chloroform, the solution was stratified, and the upper layer of the colorless aqueous phase was transferred to a freshly precooled EP tube. An equal volume of isopropanol was added slowly. The mixture was gently inverted, placed at –20°C for 30 min and centrifuged at 12,000 *g* for 10 min at 4°C. The resulting pellet was washed with 75% ethanol at a volume ratio of 1:1 with Trizol and then centrifuged again at 12,000 *g* for 10 min at 4°C. After the supernatant was discarded, the EP tube was placed on clean and dry filter paper for 5‒10 min, and the appropriate amount of nuclease-free water was added to resuspend the pellet. Then, 1–2 μL of sample was used to determine RNA concentration and purity. Reverse transcription and qPCR were performed using PrimeScript RT Master Mix (Perfect Real Time) kit (Takara, Shiga, Japan) and TB Green Premix Ex Taq (Tli RNaseH Plus) kit (Takara) according to the manufacturer’s instructions. Triplicate wells were used for each sample. The relative gene expression was calculated using the 2 ^‒∆∆Ct^ method. The primers used are listed in Table 1 .

**Table:** **Table 1** Sequences of primers used for qPCR

Gene	Forward primer (5′→3′)	Reverse primer (5′→3′)
*ACAA2*	AAGTCTCACCTGAAACAGTTGAC	CACGCAAACCAACATGCCT
*ACTB*	CTACGTCGCCCTGGACTTCGAGC	GATGGAGCCGCCGATCCACACGG

### Western blot analysis

After treatment, the cells were washed three times with PBS, and then the appropriate volume of RIPA lysis buffer (Beyotime, Shanghai, China) with protein and phosphatase inhibitor was added. The cells were lysed on ice for 30 min. The samples were transferred into a precooled EP tube and centrifuged at 13,400 *g* for 15 min at 4°C. The supernatant was collected into a new EP tube, and protein quantification was performed using a BCA kit (Beyotime) according to the instructions. Protein samples (20 μg) were subject to SDS-PAGE and then electro-transferred onto PVDF membranes (Roche, Basel, Switzerland). The membranes were blocked for 5–10 min, cut according to experimental requirements, and incubated with the corresponding diluted primary antibody ACAA2 (ab128929; Abcam, Cambridge, UK) and β-actin (#3700; CST, Beverly, USA) overnight at 4°C. The next day, the membrane strips were washed three times with TBST (containing 1% Tween 20) on a shaker, and then incubated with the HRP-conjugated secondary antibody. Finally, imaging was performed with an enhanced chemiluminescence (ECL) system (NCM Biotech, Shanghai, China)

### Data and sample collection

Gene expression data and detailed clinicopathological features of the TCGA COAD cohorts, the Gene Expression Omnibus (GEO) dataset (GSE39582), and the Oncomine database ( https://www.oncomine.com) were obtained from public databases as previously described [15]. Only patients who had complete clinical data and a follow-up of more than one month were included in the subsequent analysis. The prognostic role of ACAA2 was analyzed using a series of tissue microarrays (TMAs) developed from a cohort of 283 patients with CRC (Fudan Clinic cohort) who underwent surgery at Fudan University Zhongshan Hospital between 2015 and 2018. Information on clinicopathological characteristics and survival outcomes was gathered for all patients. Overall survival (OS) was calculated as the interval between the date of surgery and either the date of death or the date of the last follow-up. For the Fudan clinic cohort, the last follow-up date was December 30, 2019.

### Statistical analysis

The statistical analysis was performed using SPSS software version 25.0, R software version 3.6.3, and GraphPad Prism software version 7.0. All experiments were performed in at least three independent replicates. Differences between groups were analyzed using Student’s *t* test, while one-way ANOVA was used to compare multiple groups. Pearson’s chi-square test was used to evaluate the association between ACAA2 expression levels and clinicopathological features of CRC patients. Survival analysis was conducted using Kaplan-Meier curves, log-rank tests, and Cox regression models. A *P* value <0.05 was considered statistically significant.

## Results

### Increased cetuximab resistance downregulates the expressions of metabolism-related genes in CRC

**Figure 1:**
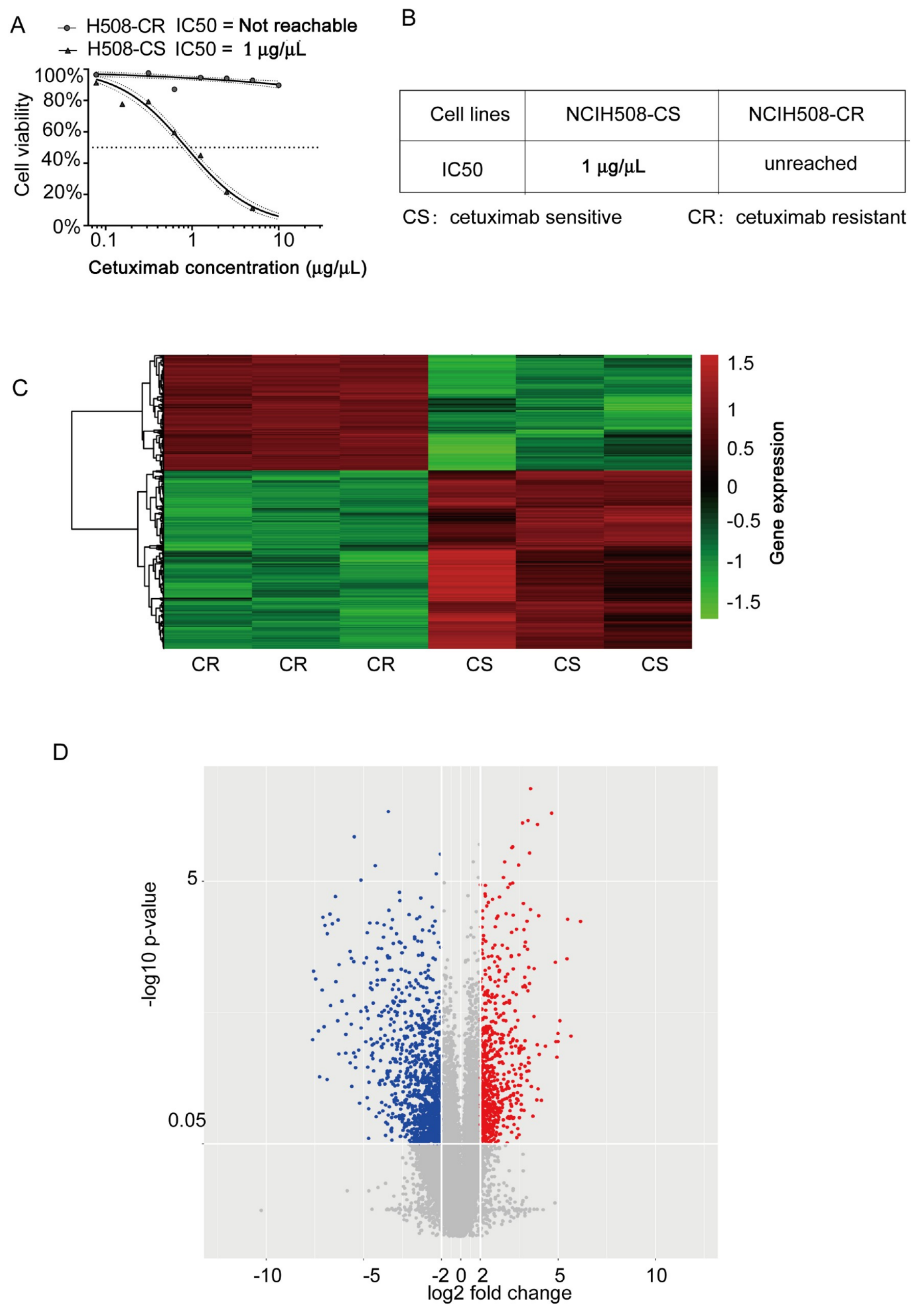
Figure 1 Distribution of differentially expressed genes between cetuximab-resistant CRC and cetuximab-sensitive CRC cells (A,B) IC50 of NCIH508 in cetuximab-sensitive (CS) and -resistant cells (CR). n=6. Data are expressed as the mean±SD. The results were obtained from three independent experiments. (C,D) Heatmap and volcano plot of differentially expressed genes between H508-CR and H508-CS (n=3). The significant difference was set at adj P<0.05 and fold change>2.

As shown in Figure 1A,B, half of NCIH508 cetuximab-sensitive cells (CS) survived in 1 μg/μL cetuximab *in vitro*. By gradually increasing the concentration of cetuximab, paired NCIH508 cetuximab-resistant cells were obtained. Screening criteria for differentially expressed genes (DEGs) were set at |logFC|>2 and adj *P*<0.05, which generated 589 upregulated genes and 891 downregulated genes. The expression patterns of these DEGs are shown in Figure 1C,D.

**Figure 2:**
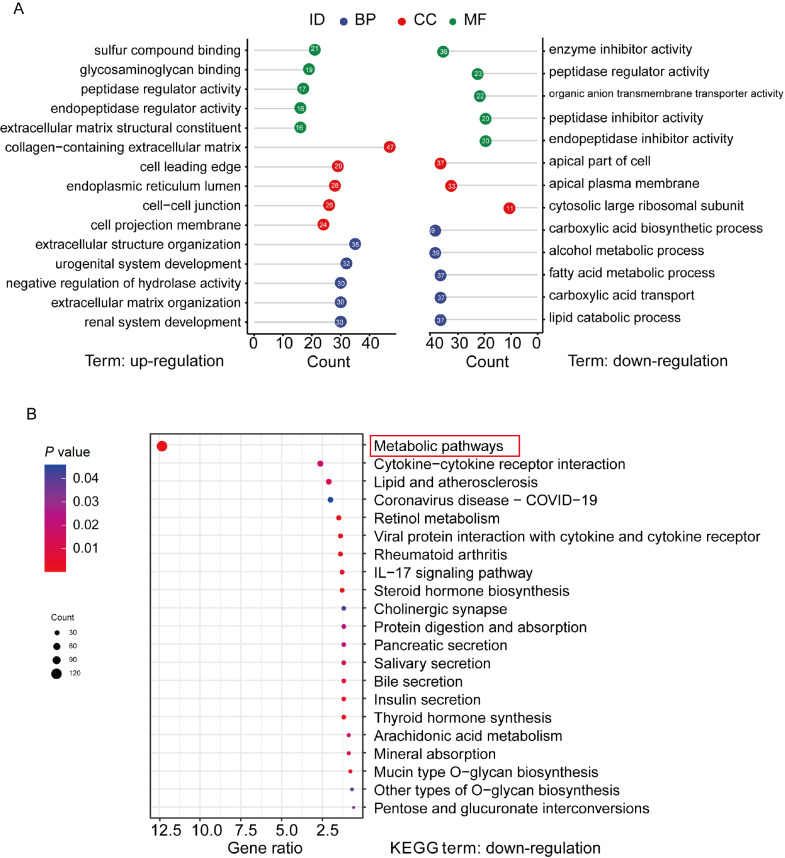
Figure 2 The functional annotation of downregulated genes between CS and CR GO enrichment analysis of DEGs between CS and CR. (B) KEGG enrichment analysis of DEGs between CS and CR. Statistical significance was set at P<0.05.

GO and KEGG analyses of those DEGs were performed ( Figure 2A,B). Both analyses revealed that the majority of downregulated genes in resistant cells were involved in metabolic processes, including lipid metabolism.

### Low ACAA2 expression is associated with cetuximab resistance in CRC

**Figure 3:**
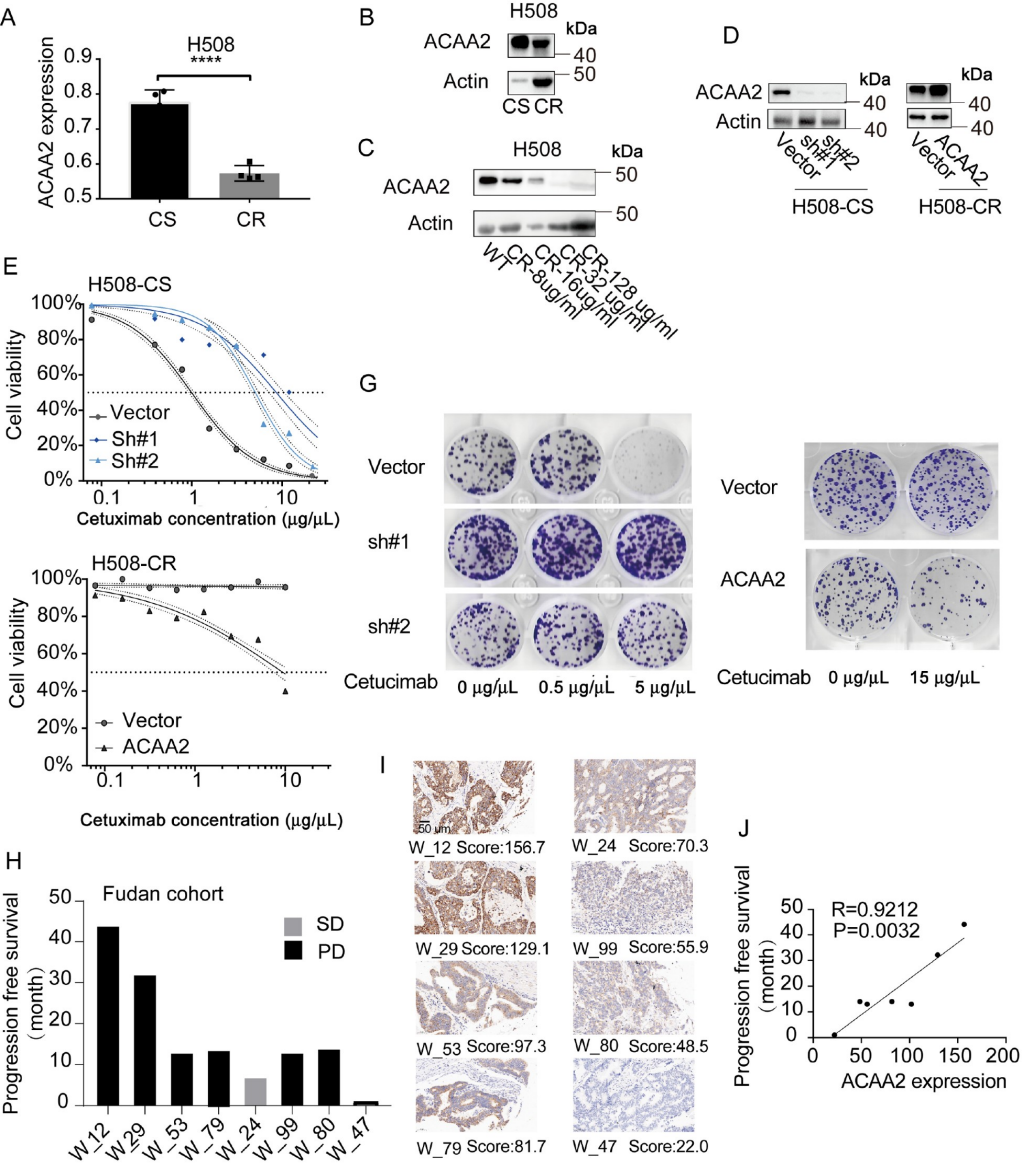
Figure 3 The relationship between ACAA2 expression and cetuximab resistance in CRC cells qPCR analysis of ACAA2 expression in CS and CR cells. n=4. Data are presented as the mean±SD. The results were from three independent experiments. (B,C) Western blot analysis of ACAA2 expression in CS and CR with different levels of cetuximab resistance. (D) The efficiency of ACAA2 knockdown and overexpression was validated by western blot analysis. Inhibition assay (E) and colony formation assay (G) for the effect of ACAA2 expression on cetuximab resistance of CS (left) and CR (right) cells. Data are shown as the mean±SD or by representative images. The results were obtained from three independent experiments. (H,I) Representative immunohistochemical staining images of ACAA2 in CRC tissues of cetuximab-treated patients (n=8; SD: stable disease, PD: progressive disease) in the Fudan cohort. (J) Pearson correlation analysis between ACAA2 expression and progression-free survival of CRC patients treated with cetuximab (n=7). ****P<0.0001; ns: no significance.

Based on our previous research [17], ACAA2 is a potential suppressor of CRC progression, but its detailed functions in CRC are still unclear. In this study, ACAA2 was considerably downregulated in CR cells ( Figure 3A,B) and progressively decreased during the development of cetuximab resistance ( Figure 3C). Previous results suggested that ACAA2 suppresses CRC progression, but the detailed mechanisms are still unclear. Lentiviruses were used for *ACAA2* knockdown in CS cells. ACAA2 was also overexpressed in CR cells ( Figure 3D). Those cells with lower ACAA2 expression had a worse response to cetuximab. *ACAA2* knockdown in HCIH508-CS cells increased the IC _50_ by 6 folds. On the other hand, ACAA2 overexpression restored the cetuximab sensitivity of NCIH508-CR cells ( Figure 3E–G). The role of ACAA2 expression in the cetuximab treatment response was validated using the Fudan cohort (8/283). Out of the eight patients, seven responded poorly to cetuximab treatment, but one responded well. The cetuximab response positively correlated with ACAA2 expression. A decrease in ACAA2 expression promoted cetuximab resistance in CRC cells. Our results suggested that a decrease in ACAA2 expression promoted cetuximab resistance in CRC cells.

### ACAA2 inhibits the proliferation of CRC cells *in vitro*

**Figure 4:**
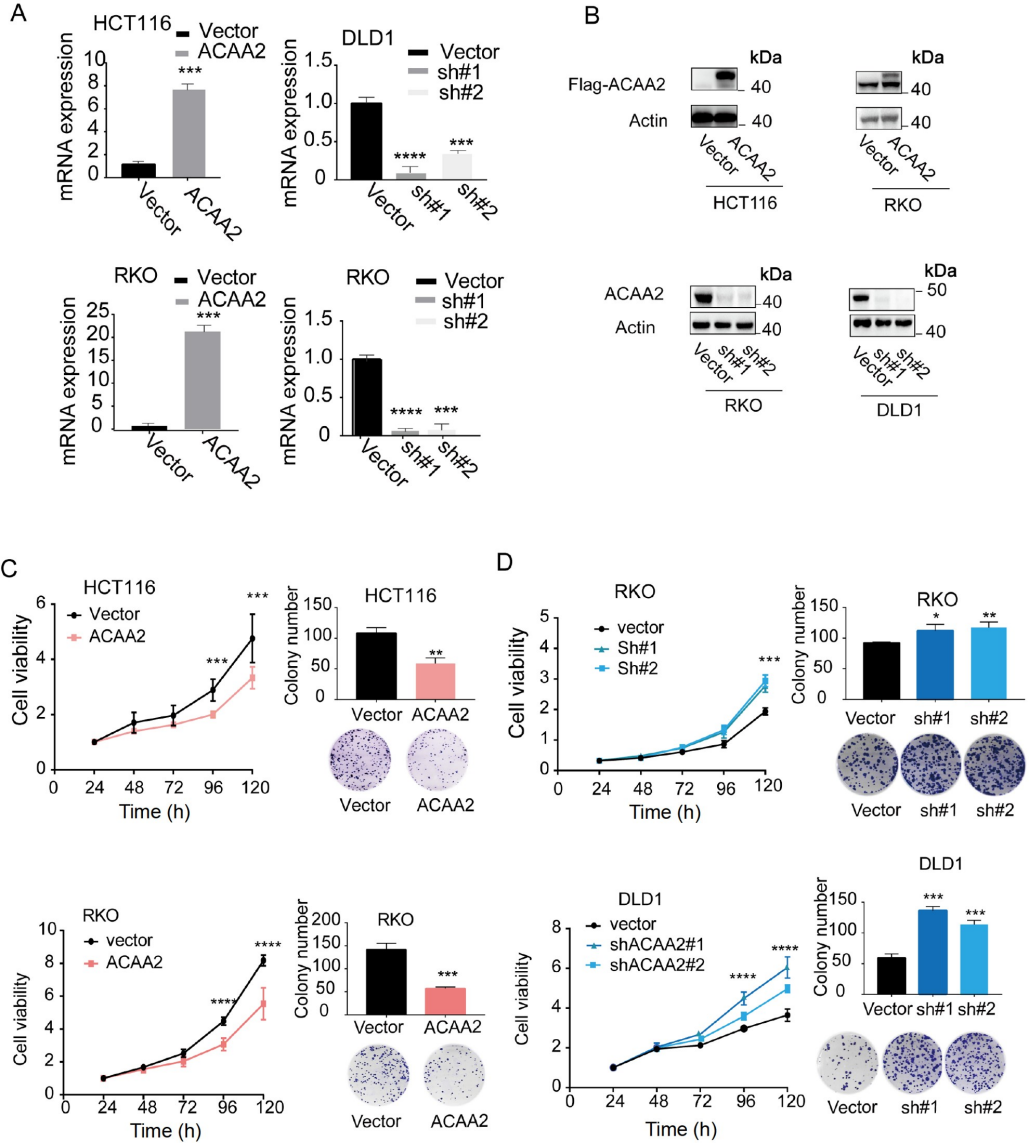
Figure 4 The effect of ACAA2 on the proliferation of CRC cells The efficiency of ACAA2 knockdown and overexpression was validated by qPCR (A) and western blot analysis (B) in CRC cells. Data are presented as the mean±SD. (C,D) CCK-8 and colony formation assays for the relationship between ACAA2 expression and proliferation of CRC cells. Differences between groups were analyzed using Student’s t test, while one-way ANOVA and Tukey’s test were used to compare multiple groups. *P<0.05; ***P<0.001; ****P<0.0001.

To explore the effects of ACAA2 on cell proliferation, stable gene editing was performed to create HCT116 and RKO cells stably overexpressing ACAA2. Stable silencing of *ACAA2* was also performed in RKO and DLD1 cells ( Figure 4A,B). CCK8 and colony formation assays revealed that ACAA2 overexpression significantly inhibited the proliferation of HCT116 ( Figure 4C, upper) and RKO ( Figure 4C, lower) cells. Conversely, *ACAA2* silencing exerted the opposite effect on the proliferation of RKO ( Figure 4D, upper) and DLD1 cells ( Figure 4D, lower).

### ACAA2 expression attenuation is related to a poor CRC outcome

**Figure 5:**
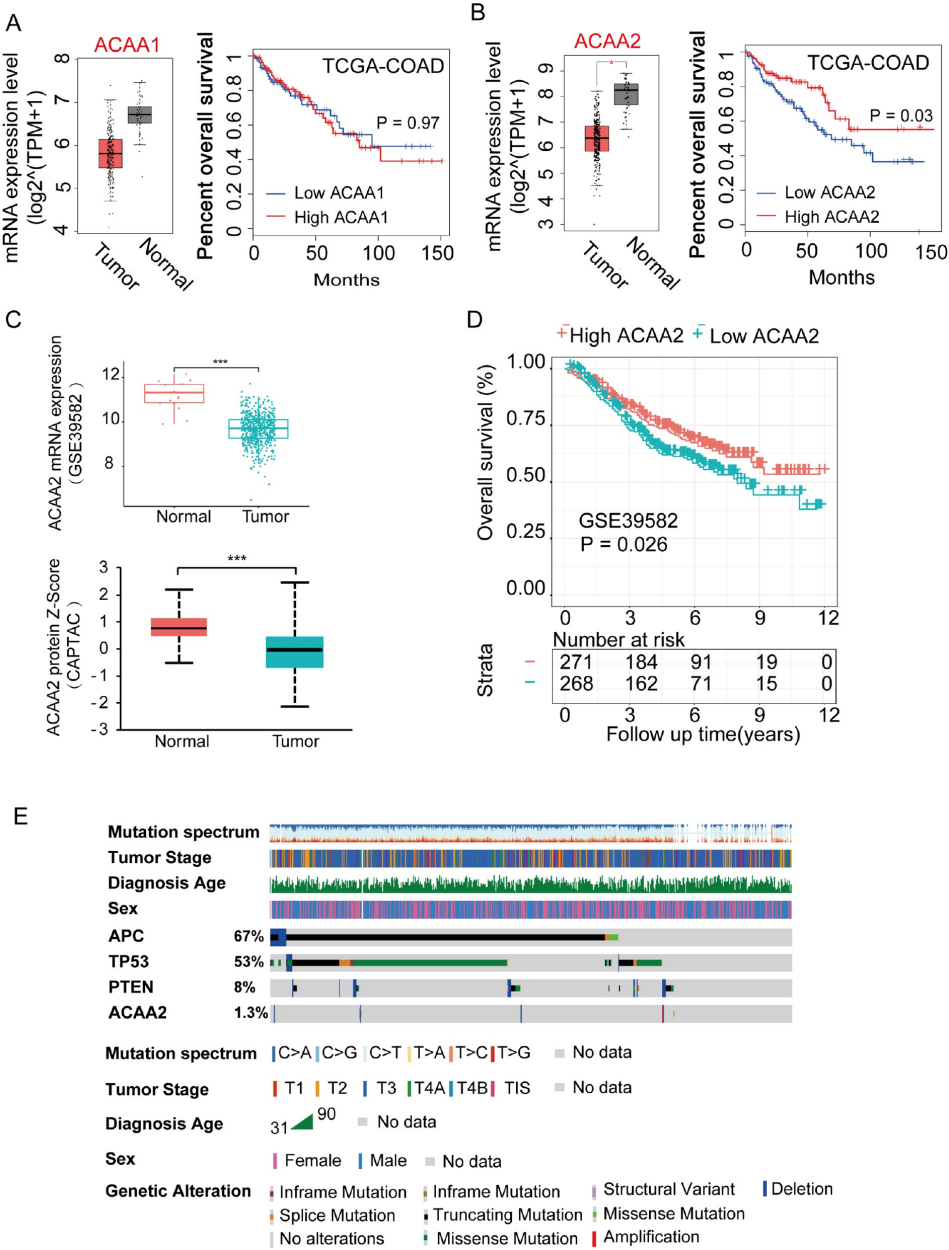
Figure 5 Patients with ACAA2 expression suffered a worse outcome (A,B) The gene expression and prognostic value of ACAA1 and ACAA2 based on the TCGA cohort (tumor: n=275, normal: n=41, patients were divided into low- and high-risk groups based on the median ACAA2 expression level). Student’s t test and a log-rank test were used to calculate the P value. (C) ACAA2 expression levels in the GSE39582 cohort (mRNA level) and CPTAC cohort (protein level). Student’s t test was used to calculate the P value. (D) Kaplan-Meier analysis and log-rank tests were used to evaluate the relationship between ACAA2 expression and OS in the GSE39582 cohort (P<0.05). (E) The clinical features and mutation patterns of ACAA2 in the TCGA cohort were analysed by cBioPortal (DNA level). *P<0.05; **P<0.01; ***P<0.001; ****P<0.0001.

As shown in Figure 5A,B, compared to that in normal tissues, ACAA2 expression was lower in CRC. Kaplan-Meier survival analysis of the TCGA cohort revealed that lower ACAA2 expression was associated with worse OS in CRC patients [hazard ratio (HR)=1.99; 95% confidence interval (CI): 1.24–3.2; *P*<0.01]. ACAA1, an isozyme of ACAA2 that works in the peroxisome [ 18, 19 ], had no similar features ( Figure 5A,B). Analysis of data in the GSE39582 and CAPTAC datasets revealed comparable findings, in which ACAA2 expression was lower in CRC than in normal tissues ( Figure 5C). Kaplan-Meier survival analysis of the GSE39582 cohort also demonstrated that CRC patients with lower ACAA2 expression had a shorter OS than those with higher ACAA2 expression [ Figure 5D; hazard ratio (HR)=1.99; 95% confidence interval (CI): 1.24–3.2; *P*<0.01]. The clinical characteristics of the TCGA and GSE39582 cohorts are shown in Supplementary Table S1.

### ACAA2 expression is related to *Kras* mutations in CRC

**Figure 6:**
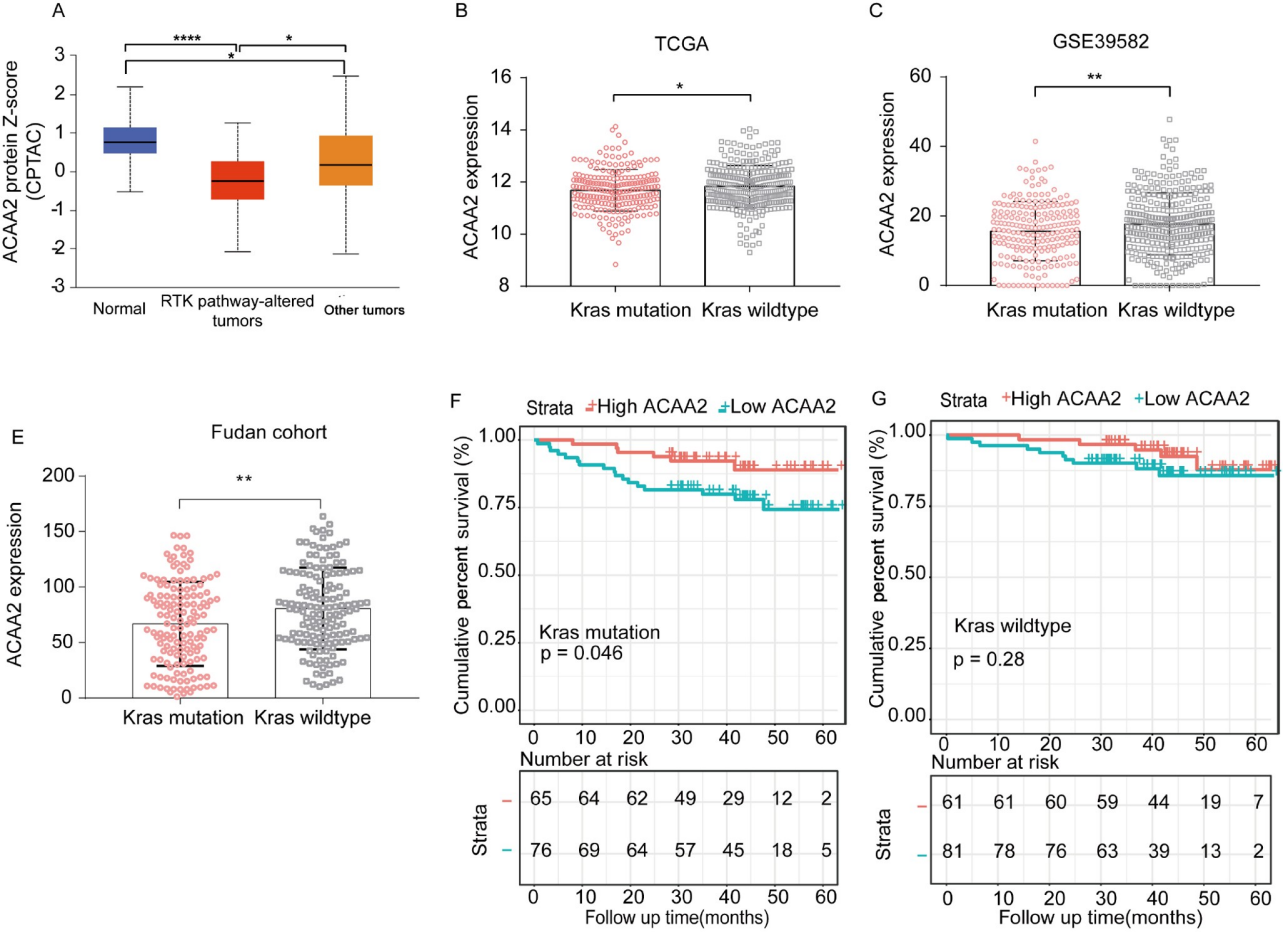
Figure 6 The relationship between ACAA2 expression and RTK-Kras signalling Expression of ACAA2 in normal tissues and tumors with different RTK signaling in the CPTAC cohort. Student’s t test was used to calculate the P value. (B) The expression of ACAA2 in tumor tissues of CRC patients with or without Kras mutation. TCGA cohort data were used. Student’s t test was used to calculate the P value. (C) The difference in the expression of ACAA2 in tumor tissues of CRC patients with or without Kras mutation in the GSE39582 cohort. Student’s t test was used to calculate the P value. (E) The difference in the expression of ACAA2 in tumor tissues of CRC patients with or without Kras mutation in the Fudan clinic cohort. Student’s t test was used to calculate the P value. (F,G) Prognostic value of ACAA2 in CRC patients with or without Kras mutation in the Fudan clinical cohort. Kaplan-Meier analysis and log-rank tests were used to calculate the P value. *P<0.05; **P<0.01; ***P<0.001; ****P <0.0001.

Lower mRNA expression can arise from mutations in inactive DNA or transcriptional suppression. Analysis of the gene mutation spectrum revealed that APC and TP53 are the two most commonly mutated genes in colorectal cancer, whereas PTEN is the most frequently mutated suppressor gene. A deletion mutation in ACAA2 was observed in patients in the TCGA cohort ( Figure 5E). Altered RTK-Kras signaling is a primary reason for cetuximab resistance [ 20, 21]. Interestingly, the results showed that CRC patients with impaired receptor tyrosine kinase (RTK) pathway signaling displayed lower ACAA2 expression than those without impaired RTK ( Figure 6A). In the TCGA cohort ( Figure 6B), 222 (42.3%) CRC patients had *Kras* mutations, while 303 (57.7%) CRC patients had *Kras* wild-type genes. In GSE39582 ( Figure 6C), there were 213 (39.8%) CRC patients with *Kras* mutations and 322 (60.2%) with *Kras* wild-type genes. In both cohorts, ACAA2 expression was lower in CRC patients with *Kras* mutations than in those without *Kras* mutations ( *P*<0.05).

A Fudan clinical cohort that included 141 (49.8%) patients with *Kras* mutations and 142 (50.2%) patients with wild-type Kras was enrolled, and their clinical characteristics at baseline are shown in Supplementary Tables S2 and S3, respectively. Further analysis revealed that ACAA2 expression was significantly lower in patients with *Kras* mutation ( Figure 6D). Patients could be stratified into poor or favourable OS based on ACAA2 expression, and the stratification was more accurate in patients with *Kras* mutations ( Figure 6F–G).

Univariate and multivariate Cox analyses were performed to confirm the prognostic value of ACAA2 for CRC patients ( Tables 2 and 3). In CRC patients with *Kras* mutations, the risk ratio of death was 7.04-fold higher in patients with late-stage disease than in patients with early-stage disease ( *P*=0.002). Patients with lower ACAA2 expression had a 2.43-fold higher risk of death than those with higher ACAA2 expression ( *P*=0.043). In contrast, colorectal cancer patients with Kras wild-type had a 4.6-fold greater risk of mortality than those with earlier stage disease. While not statistically significant, patients with lower ACAA2 expression levels tended to have poorer outcomes. Therefore, we speculate that low ACAA2 expression might be related to *Kras* mutation and CRC prognosis.

**Table:** **Table 2** Univariate and multivariate analysis of factors associated with OS in the Fudan clinic cohort with *Kras* mutation

Variables		Univariate analysis HR [CI]	*P* value	Multivariate analysis HR [CI]	*P* value
Sex	male	Reference		Reference	
	female	1.20 [0.53–2.75]	0.66	0.96 [0.42–2.19]	0.914
Age	<65	Reference		Reference	
	≥65	2.01 [0.87–4.66]	0.102	2.15 [0.91–5.08]	0.083
Location	left	Reference		Reference	
	right	0.83 [0.36–1.89]	0.652	0.90 [0.39–2.08]	0.802
Stage	I+II	Reference		Reference	
	III+IV	6.52 [1.94–21.96]	**0.002**	7.04 [2.08–23.81]	**0.002**
ACAA2	high	Reference		Reference	
	low	2.57 [1.01–6.52]	**0.047**	2.43 [0.95–6.23]	**0.043**

OS, overall survival; HR, hazard ratio; CI, confidential interval. Data were obtained from the Cox proportional hazards model. *P*<0.05 was regarded as statistically significant, which was shown in bold.

**Table:** **Table 3** Univariate and multivariate analysis of factors associated with OS in the Fudan clinic cohort with Kras wild-type

Variables		Univariate analysis HR [CI]	*P* value	Multivariate analysis HR [CI]	*P* value
Sex	male	Reference		Reference	
	female	1.99 [0.68–5.82]	0.210	2.18 [0.71–6.74]	0.174
Age	<65	Reference		Reference	
	≥65	2.68 [0.91–7.87]	0.072	4.41 [1.30–14.93]	**0.017**
Location	left	Reference		Reference	
	right	0.78 [0.28–2.16]	0.635	1.18 [0.38–3.62]	0.775
Stage	I+II	Reference		Reference	
	III+IV	2.71 [0.86–8.52]	0.088	4.66 [1.40–15.48]	**0.012**
ACAA2	high	Reference		Reference	
	low	1.79 [0.61–5.26]	0.289	1.48 [0.49–4.48]	0.488

OS, overall survival; HR, hazard ratio; CI, confidential interval. Data were obtained from the Cox proportional hazards model. *P*<0.05 was regarded as statistically significant, which was shown in bold.

## Discussion

Activation of RTK signaling, such as EGFR, insulin-like growth factor receptor (IGFR), and hepatocyte growth factor (HGF), contributes to the survival and progression of CRC [ 22– 24]. Anti-EGFR therapy targets EGFR on the cell membrane and inhibits downstream RTK signaling [25]. However, resistance to anti-EGFR therapy in CRC is a complex process involving numerous genes and other variables [26]. For instance, *Ras* mutations lead CRC patients to be primary resistant to this treatment via direct downstream hyperactive signaling [27]. Individuals with wild-type Ras may generally gain secondary resistance after a period of treatment [ 6, 28, 29]. Cetuximab treatment is one of the important components of anti-EGFR treatment in the clinical management of CRC, but it is only ideal in patients with wild-type Ras/Raf [30]. Unfortunately, the duration of response is also affected by acquired drug resistance from gene mutations, such as mutations in the *Kras* gene [31], and other transcriptomic mechanisms [ 8, 32, 33].

In this study, CRC cell lines were exposed to increasing concentrations of cetuximab to induce cetuximab resistance. RNA sequencing analysis revealed that downregulated genes significantly participated in metabolic processes, suggesting that a metabolic disorder occurs in CRC during the development of cetuximab resistance, consistent with previous reports. For example, studies have shown that methylglyoxal (MGO), a glycolysis byproduct, can induce cetuximab resistance in CRC cells under stress stimulation [9]. Furthermore, by interacting with phosphofructokinase 1, TRAP1 improves Warburg metabolism and promotes resistance to cetuximab treatment in CRC [7].

Metabolic disorders are generally associated with dysregulated expressions of metabolism-related genes (MRGs) [ 34, 35]. Previous studies have shown that these genes regulate the complex progression of CRC [ 36, 37] and are useful prognostic biomarkers [17] and prospective therapeutic targets [ 33, 38] for CRC management. In this study, we observed a gradual reduction in the expression of *ACAA2*, a metabolism-related gene, during the transformation of a cetuximab-sensitive cell line into its resistant type. *ACAA2* knockout induced cetuximab sensitivity and promoted the proliferation of CRC cells, suggesting that ACAA2 suppressed CRC progression in various ways. One potential mechanism by which ACAA2 affects cetuximab resistance is through direct cleavage by PRSS1 [39]. However, this mechanism may only explain the effect of ACAA2 on drug resistance, and further research is needed to fully understand its role in CRC progression.

A positive correlation was found between ACAA2 expression and the duration of responses in some patients undergoing cetuximab treatment. However, this result needs to be validated. Furthermore, the data revealed that a decrease in ACAA2 expression was more likely resulted from transcriptional regulation rather than genetic deletion, which occurred at a very low frequency. Since the downregulation of ACAA2 expression occurred at both the mRNA and protein levels, transcriptional regulation is likely to be the primary mechanism underlying the downregulation of ACAA2 expression. Future studies should focus on uncovering the molecular mechanism that regulates ACAA2 expression in CRC.

Both primary and secondary cetuximab resistance in CRC and head and neck squamous cell carcinoma are associated with the upregulation and activation of the RTK pathway [ 40 – 44]. The findings suggest that ACAA2 is significantly lower in CRC tumours with dysregulated RTK expression than in those without, highlighting a potential correlation between ACAA2 and RTK expression.

Kras is a crucial downstream effector of RTK signaling and serves as a critical clinical indicator for cetuximab therapy response in CRC [45]. It plays an important role in driving cetuximab resistance [8], promoting unlimited cell proliferation [46] and disrupting serious metabolic homeostasis [ 47‒ 49]. Correcting these metabolic disorders may reverse malignant cellular transformation caused by *Kras* mutation. For instance, removing pyruvate, a key glycolytic metabolite, can significantly enhance the sensitivity of colon cancer cells with *Kras* mutation to cetuximab [9]. Inhibiting aspartate synthase activity and glutamine uptake reduces glutamine metabolism-dependent tumour growth in tumours with *Kras* mutation [ 50, 51]. These findings suggest that Kras may induce EGFR monoclonal resistance in CRC by affecting energy metabolism. Therefore, the correlation between ACAA2 expression and *Kras* mutation was explored further. Intriguingly, the results indicated that ACAA2 expression is downregulated in CRC cells with *Kras* mutation. Moreover, its prognostic value was more significant in patients with *Kras* mutations. The role of ACAA2 was disrupted in the wild-type *Kras* gene, which may be due to cetuximab treatment during tumor progression. ACAA2 may contribute to both primary and secondary resistance to cetuximab therapy in patients with *Kras* mutations, but the underlying molecular mechanisms require further investigation.

In conclusion, our findings suggest that metabolism-related genes, such as *ACAA2*, play important roles in the development of cetuximab resistance in CRC cells. The downregulation of ACAA2 expression may be linked to *Kras* mutations. These results provide new insights into the metabolic basis of cetuximab resistance in CRC and may contribute to the discovery of metabolic checkpoints and the development of novel therapeutic strategies for CRC treatment patients with cetuximab resistance. Further investigation is needed to elucidate the biological mechanisms and pathways involved in CRC resistance to cetuximab.

## Supporting information

23066Supplementary_Tables

## Supplementary Data

Supplementary data is available at *Acta Biochimica et Biophysica Sinica* online.

## References

[1] Sung H, Ferlay J, Siegel RL, Laversanne M, Soerjomataram I, Jemal A, Bray F (2021). Global cancer statistics 2020: GLOBOCAN estimates of incidence and mortality worldwide for 36 cancers in 185 countries. CA Cancer J Clin.

[2] Zhang R, Liu S, Gong B, Xie W, Zhao Y, Xu L, Zheng Y (2022). Kif4A mediates resistance to neoadjuvant chemoradiotherapy in patients with advanced colorectal cancer via regulating DNA damage response. Acta Biochim Biophys Sin.

[3] Du L, Liu N, Jin J, Cao M, Sun Y, Gao X, Ruan B (2022). ZNF3 regulates proliferation, migration and invasion through MMP1 and TWIST in colorectal cancer. Acta Biochim Biophys Sin.

[4] Holch JW, Ricard I, Stintzing S, Modest DP, Heinemann V (2017). The relevance of primary tumour location in patients with metastatic colorectal cancer: a meta-analysis of first-line clinical trials. Eur J Cancer.

[5] Porru M, Pompili L, Caruso C, Biroccio A, Leonetti C (2018). Targeting KRAS in metastatic colorectal cancer: current strategies and emerging opportunities. J Exp Clin Cancer Res.

[6] Diaz Jr LA, Williams RT, Wu J, Kinde I, Hecht JR, Berlin J, Allen B (2012). The molecular evolution of acquired resistance to targeted EGFR blockade in colorectal cancers. Nature.

[7] Maddalena F, Condelli V, Matassa DS, Pacelli C, Scrima R, Lettini G, Li Bergolis V (2020). TRAP1 enhances Warburg metabolism through modulation of PFK1 expression/activity and favors resistance to EGFR inhibitors in human colorectal carcinomas. Mol Oncol.

[8] Misale S, Yaeger R, Hobor S, Scala E, Janakiraman M, Liska D, Valtorta E (2012). Emergence of KRAS mutations and acquired resistance to anti-EGFR therapy in colorectal cancer. Nature.

[9] Bellier J, Nokin MJ, Caprasse M, Tiamiou A, Blomme A, Scheijen JL, Koopmansch B (2020). Methylglyoxal Scavengers Resensitize KRAS-mutated colorectal tumors to cetuximab. Cell Rep.

[10] Najumudeen AK, Ceteci F, Fey SK, Hamm G, Steven RT, Hall H, Nikula CJ (2021). The amino acid transporter SLC7A5 is required for efficient growth of KRAS-mutant colorectal cancer. Nat Genet.

[11] Cohen AS, Geng L, Zhao P, Fu A, Schulte ML, Graves-Deal R, Washington MK (2020). Combined blockade of EGFR and glutamine metabolism in preclinical models of colorectal cancer. Transl Oncol.

[12] Berger AK, Lücke S, Abel U, Haag GM, Grüllich C, Stange A, Dietrich M (2018). Early metabolic response in sequential FDG-PET/CT under cetuximab is a predictive marker for clinical response in first-line metastatic colorectal cancer patients: results of the phase II REMOTUX trial. Br J Cancer.

[13] Wu C, Song H, Fu X, Li S, Jiang T (2020). Transcriptomic analysis of glioma based on IDH status identifies ACAA2 as a prognostic factor in lower grade glioma. Biomed Res Int.

[14] Choi S, Pfleger J, Jeon YH, Yang Z, He M, Shin H, Sayed D (2019). Oxoglutarate dehydrogenase and acetyl-CoA acyltransferase 2 selectively associate with H2A.Z-occupied promoters and are required for histone modifications. Biochim Biophys Acta (BBA)-Gene Regulatory Mech.

[15] Sheng X, Cristea IM. The antiviral sirtuin 3 bridges protein acetylation to mitochondrial integrity and metabolism during human cytomegalovirus infection.
PLoS Pathog 2021, 17: e1009506. https://doi.org/10.1371/journal.ppat.1009506.

[16] Kodama T, Bard-Chapeau EA, Newberg JY, Kodama M, Rangel R, Yoshihara K, Ward JM (2016). Two-step forward genetic screen in mice identifies ral GTPase-activating proteins as suppressors of hepatocellular carcinoma. Gastroenterology.

[17] Yuan Y, Liu M, Hou P, Liang L, Sun X, Gan L, Liu T (2021). Identification of a metabolic signature to predict overall survival for colorectal cancer. Scand J Gastroenterol.

[18] Bout A, Teunissen Y, Hashimoto T, Benne R, Tager JM (1988). Nucleotide sequence of human peroxisomal 3-oxoacyl-CoA thiolase. Nucleic Acids Res.

[19] Abe H, Ohtake A, Yamamoto S, Satoh Y, Takayanagi M, Amaya Y, Takiguchi M (1993). Cloning and sequence analysis of a full length cDNA encoding human mitochodrial 3-oxoacyl-CoA thiolase. Biochim Biophys Acta.

[20] Leonard B, Brand TM, O′Keefe RA, Lee ED, Zeng Y, Kemmer JD, Li H (2018). BET inhibition overcomes receptor tyrosine kinase-mediated cetuximab resistance in HNSCC. Cancer Res.

[21] Lièvre A, Laurent-Puig P (2009). Predictive value of KRAS mutations in chemoresistant CRC. Nat Rev Clin Oncol.

[22] Vitiello PP, Cardone C, Martini G, Ciardiello D, Belli V, Matrone N, Barra G (2019). Receptor tyrosine kinase-dependent PI3K activation is an escape mechanism to vertical suppression of the EGFR/RAS/MAPK pathway in KRAS-mutated human colorectal cancer cell lines. J Exp Clin Cancer Res.

[23] Yoshihiro T, Ariyama H, Yamaguchi K, Imajima T, Yamaga S, Tsuchihashi K, Isobe T (2022). Inhibition of insulin‐like growth factor‐1 receptor enhances eribulin-induced DNA damage in colorectal cancer. Cancer Sci.

[24] Joosten SPJ, Spaargaren M, Clevers H, Pals ST (2020). Hepatocyte growth factor/MET and CD44 in colorectal cancer: partners in tumorigenesis and therapy resistance. Biochim Biophys Acta Rev Cancer.

[25] Li S, Schmitz KR, Jeffrey PD, Wiltzius JJW, Kussie P, Ferguson KM (2005). Structural basis for inhibition of the epidermal growth factor receptor by cetuximab. Cancer Cell.

[26] Martini G, Ciardiello D, Vitiello PP, Napolitano S, Cardone C, Cuomo A, Troiani T (2020). Resistance to anti-epidermal growth factor receptor in metastatic colorectal cancer: what does still need to be addressed?. Cancer Treatment Rev.

[27] Normanno N, Tejpar S, Morgillo F, De Luca A, Van Cutsem E, Ciardiello F (2009). Implications for KRAS status and EGFR-targeted therapies in metastatic CRC. Nat Rev Clin Oncol.

[28] Pozzi C, Cuomo A, Spadoni I, Magni E, Silvola A, Conte A, Sigismund S (2016). The EGFR-specific antibody cetuximab combined with chemotherapy triggers immunogenic cell death. Nat Med.

[29] Pietrantonio F, Vernieri C, Siravegna G, Mennitto A, Berenato R, Perrone F, Gloghini A (2017). Heterogeneity of acquired resistance to Anti-EGFR monoclonal antibodies in patients with metastatic colorectal cancer. Clin Cancer Res.

[30] Sorich MJ, Wiese MD, Rowland A, Kichenadasse G, McKinnon RA, Karapetis CS (2015). Extended RAS mutations and anti-EGFR monoclonal antibody survival benefit in metastatic colorectal cancer: a meta-analysis of randomized, controlled trials. Ann Oncol.

[31] Johnson RM, Qu X, Lin CF, Huw LY, Venkatanarayan A, Sokol E, Ou FS (2022). ARID1A mutations confer intrinsic and acquired resistance to cetuximab treatment in colorectal cancer. Nat Commun.

[32] Woolston A, Khan K, Spain G, Barber LJ, Griffiths B, Gonzalez-Exposito R, Hornsteiner L (2019). Genomic and transcriptomic determinants of therapy resistance and immune landscape evolution during anti-EGFR treatment in colorectal cancer. Cancer Cell.

[33] Hong HJ, Shao Y, Zhang S, Yang G, Jia H, Yang X, Huang L (2022). ACACB is a novel metabolism-related biomarker in the prediction of response to cetuximab therapy inmetastatic colorectal cancer. Acta Biochim Biophys Sin.

[34] Claussnitzer M, Susztak K (2021). Gaining insight into metabolic diseases from human genetic discoveries. Trends Genet.

[35] Balliu B, Carcamo-Orive I, Gloudemans MJ, Nachun DC, Durrant MG, Gazal S, Park CY (2021). An integrated approach to identify environmental modulators of genetic risk factors for complex traits. Am J Hum Genet.

[36] Blaha CS, Ramakrishnan G, Jeon SM, Nogueira V, Rho H, Kang S, Bhaskar P (2022). A non-catalytic scaffolding activity of hexokinase 2 contributes to EMT and metastasis. Nat Commun.

[37] Fan M, Sun W, Gu X, Lu S, Shen Q, Liu X, Zhang X (2022). The critical role of STAT3 in biogenesis of tumor-derived exosomes with potency of inducing cancer cachexia in vitro and in vivo. Oncogene.

[38] Huang Y, Zhou J, Luo S, Wang Y, He J, Luo P, Chen Z (2018). Identification of a fluorescent small-molecule enhancer for therapeutic autophagy in colorectal cancer by targeting mitochondrial protein translocase TIM44. Gut.

[39] Tan Z, Gao L, Wang Y, Yin H, Xi Y, Wu X, Shao Y (2020). PRSS contributes to cetuximab resistance in colorectal cancer. Sci Adv.

[40] Wheeler DL, Huang S, Kruser TJ, Nechrebecki MM, Armstrong EA, Benavente S, Gondi V (2008). Mechanisms of acquired resistance to cetuximab: role of HER (ErbB) family members. Oncogene.

[41] Hu S, Dai H, Li T, Tang Y, Fu W, Yuan Q, Wang F (2016). Broad RTK-targeted therapy overcomes molecular heterogeneity-driven resistance to cetuximab via vectored immunoprophylaxis in colorectal cancer. Cancer Lett.

[42] Cardone C, Blauensteiner B, Moreno-Viedma V, Martini G, Simeon V, Vitiello PP, Ciardiello D (2020). AXL is a predictor of poor survival and of resistance to anti-EGFR therapy in RAS wild-type metastatic colorectal cancer. Eur J Cancer.

[43] Madoz-Gúrpide J, Zazo S, Chamizo C, Casado V, Caramés C, Gavín E, Cristóbal I (2015). Activation of MET pathway predicts poor outcome to cetuximab in patients with recurrent or metastatic head and neck cancer. J Transl Med.

[44] Brand TM, Iida M, Stein AP, Corrigan KL, Braverman CM, Luthar N, Toulany M (2014). AXL mediates resistance to cetuximab therapy. Cancer Res.

[45] Young A, Lou D, McCormick F (2013). Oncogenic and wild-type Ras play divergent roles in the regulation of mitogen-activated protein kinase signaling. Cancer Discov.

[46] Luo Y, Li Z, Kong Y, He W, Zheng H, An M, Lin Y (2022). KRAS mutant–driven SUMOylation controls extracellular vesicle transmission to trigger lymphangiogenesis in pancreatic cancer. J Clin Invest.

[47] Son J, Lyssiotis CA, Ying H, Wang X, Hua S, Ligorio M, Perera RM (2013). Glutamine supports pancreatic cancer growth through a KRAS-regulated metabolic pathway. Nature.

[48] Amendola CR, Mahaffey JP, Parker SJ, Ahearn IM, Chen WC, Zhou M, Court H (2019). KRAS4A directly regulates hexokinase 1. Nature.

[49] Cenigaonandia-Campillo A, Serna-Blasco R, Gómez-Ocabo L, Solanes-Casado S, Baños-Herraiz N, Puerto-Nevado LD, Cañas JA (2021). Vitamin C activates pyruvate dehydrogenase (PDH) targeting the mitochondrial tricarboxylic acid (TCA) cycle in hypoxic
*KRAS* mutant colon cancer. Theranostics.

[50] Hao Y, Samuels Y, Li Q, Krokowski D, Guan BJ, Wang C, Jin Z (2016). Oncogenic PIK3CA mutations reprogram glutamine metabolism in colorectal cancer. Nat Commun.

[51] Toda K, Kawada K, Iwamoto M, Inamoto S, Sasazuki T, Shirasawa S, Hasegawa S (2016). Metabolic alterations caused by KRAS mutations in colorectal cancer contribute to cell adaptation to glutamine depletion by upregulation of asparagine synthetase. Neoplasia.

