# Supplementary material for: Negative correlation between acetyl-CoA acyltransferase 2 and cetuximab resistance in colorectal cancer: Correlation between ACAA2 and cetuximab resistance in CRC
Source: Acta Biochim Biophys Sin (Shanghai). 2023 Jun 13;55(9):1467–78. doi: 10.3724/abbs.2023111 (PMC10520478; doi:10.3724/abbs.2023111)
Supplement: 23066Supplementary_Tables [file 23066Supplementary_Tables.pdf]

**Supplementary Table S1. Patients and tumor clinicopathological characteristics of TCGA and GSE39582 datasets**

| Variable | TCGA overall | ACAA high | ACAA low | *P* | GEO overall | ACAA high | ACAA low | *P* |
|---|---|---|---|---|---|---|---|---|
| N | 525 (100%) | 261 (49.7%) | 264 (50.3%) | | 510 (100%) | 269 (52.7%) | 241 (47.3%) | |
| Age | | | | | | | | |
| <60 | 175 (33.3%) | 84 (31.8%) | 91 (34.5%) | 0.276 | 140 (27.5%) | 74 (27.5%) | 66 (27.4%) | 0.975 |
| ≥60 | 350 (66.7%) | 177 (67.0%) | 173 (65.5%) | | 370 (72.5%) | 195 (72.5%) | 175 (72.6%) | |
| NA | 0 (0%) | 3 (1.1%) | 0 (0%) | | | | | |
| Gender | | | | | | | | |
| Male | 275 (52.4%) | 125 (47.3%) | 139 (52.7%) | 0.579 | 277 (54.3%) | 146 (45.7%) | 131 (54.4%) | 0.985 |
| Female | 250 (47.6%) | 136 (51.5%) | 125 (47.3%) | | 233 (45.7%) | 123 (54.3%) | 110 (45.6%) | |
| Stage | | | | | | | | |
| I | 91 (17.3%) | 53 (20.1%) | 38 (14.4%) | | 37 (7.3%) | 26 (9.7%) | 11 (4.6%) | |
| II | 195 (37.1%) | 101 (38.3%) | 94 (35.6%) | 0.158 | 246 (48.2%) | 126 (46.8%) | 120 (49.8%) | **0.026** |
| III | 160 (30.5%) | 71 (26.9%) | 89 (33.7%) | | 167 (32.7%) | 93 (34.6%) | 74 (30.7%) | |
| IV | 78 (14.9%) | 36 (13.6%) | 42 (15.9%) | | 60 (11.8%) | 24 (8.9%) | 36 (14.9%) | |
| NA | 0 (0%) | 3 (1.1%) | 1 (0.4%) | | 0 (0%) | 0 (0%) | 0 (0%) | |
| MMR | | | | | | | | |
| dMMR | 71 (13.5%) | 28 (10.6%) | 43 (16.3%) | | 72 (14.1%) | 37 (13.8%) | 35 (14.5%) | |
| pMMR | 428 (81.5%) | 218 (82.6%) | 210 (79.5%) | 0.073 | 391 (76.7%) | 209 (77.7%) | 182 (75.5%) | 0.747 |
| NA | 26 (5.0%) | 15 (5.7%) | 11 (4.2%) | | 47 (9.2%) | 23 (8.6%) | 24 (10%) | |
| Location | | | | | | | | |
| Left | 279 (53.1%) | 133 (50.4%) | 146 (55.3%) | | 303 (59.4%) | 157 (58.4%) | 146 (60.6%) | |

| | | | | | | | | |
|---|---|---|---|---|---|---|---|---|
| Right | 225 (42.9%) | 118 (44.7%) | 107 (40.5%) | 0.287 | 207 (40.6%) | 112 (41.6%) | 95 (39.4%) | 0.611 |
| NA | 21 (4.0%) | 0 (0%) | 0 (0%) | | 0 (0%) | 0 (0%) | 0 (0%) | |
| TP53 | | | | | | | | |
| Mutate | 317 (60.4%) | 141 (53.4%) | 176 (66.7%) | | 169 (33.1%) | 81 (30.1%) | 88 (36.5%) | |
| Wild Type | 0 (0%) | 0 (0%) | 0 (0%) | NA | 146 (28.6%) | 88 (32.7%) | 58 (24.1%) | **0.028** |
| NA | 208 (39.6%) | 120 (45.5%) | 88 (33.3%) | | 169 (38.2%) | 100 (37.2%) | 95 (39.4%) | |
| KRAS | | | | | | | | |
| Mutate | 222 (42.3%) | 102 (38.6%) | 120 (45.5%) | | 204 (40%) | 101 (37.5%) | 103 (42.7%) | |
| Wild type | 303 (57.7%) | 159 (60.2%) | 144 (54.5%) | 0.139 | 306 (60%) | 168 (62.5%) | 138 (57.3%) | 0.232 |
| NA | 0 (0%) | 0 (0%) | 0 (0%) | | 0 (0%) | 0 (0%) | 0 (0%) | |
| BRAF | | | | | | | | |
| Mutate | 64 (12.2%) | 31 (11.7%) | 33 (12.5%) | | 51 (10.0%) | 27 (10%) | 24 (10%) | |
| Wild type | 461 (87.8%) | 230 (87.1%) | 231 (87.5%) | 0.827 | 459 (90%) | 242 (90%) | 217 (90%) | 0.976 |
| NA | 0 (0%) | 0 (0%) | 0 (0%) | | 0 (0%) | 0 (0%) | 0 (0%) | |
| CMS | | | | | | | | |
| CMS1 | 62 (11.8%) | 28 (10.6%) | 34 (12.9%) | | 86 (16.9%) | 44 (16.4%) | 42 (17.4%) | |
| CMS2 | 177 (33.7%) | 95 (36.0%) | 82 (31.1%) | | 204 (40.0%) | 114 (42.3%) | 90 (37.3%) | |
| CMS3 | 61 (11.6%) | 34 (12.9%) | 27 (10.2%) | 0.362 | 65 (12.7%) | 32 (11.9%) | 33 (13.7%) | **< 0.001** |
| CMS4 | 124 (23.6%) | 55 (20.8%) | 69 (26.1%) | | 113 (22.2%) | 46 (17.1%) | 67 (27.8%) | |
| NA | 101 (19.1%) | 49 (18.6%) | 52 (19.7%) | | 42 (8.2%) | 33 (12.3%) | 9 (3.7%) | |

N: number; NA: no available; $P < 0.05$ was considered statistically significant.

**Supplementary Table S2. Patients and tumor clinicopathological characteristics of the Fudan clinic cohort with KRAS mutation**

| Characteristics | | All | ACAA2 high | ACAA2 low |
|---|---|---|---|---|
| N | | 141 | 65 | 76 |
| Sex (%) | Female | 72 (51.1) | 32 (48.5) | 40 (53.3) |
| | Male | 69 (48.9) | 34 (51.5) | 35 (46.7) |
| Age (%) | >=65 | 64 (45.4) | 30 (45.5) | 34 (45.3) |
| | <65 | 77 (54.6) | 36 (54.5) | 41 (54.7) |
| Location (%) | Left | 73 (51.8) | 36 (54.5) | 37 (49.3) |
| | Right | 66 (46.8) | 30 (45.5) | 36 (48.0) |
| | NA | 2 (1.4) | 0 (0.0) | 12 (2.7) |
| TNM stage (%) | I | 18 (12.8) | 10 (15.2) | 8 (10.7) |
| | II | 47 (33.3) | 21 (31.8) | 26 (34.7) |
| | III | 53 (37.6) | 23 (34.8) | 30 (40.0) |
| | IV | 23 (16.3) | 12 (18.2) | 11 (14.7) |

N/n, number; T, tumor; N, Lymphnodes; M, metastasis; MMR, mismatch repair.

**Supplementary Table S3. Patients and tumor clinicopathological characteristics of the Fudan clinic cohort with KRAS wild-type**

| Characteristics | | All | ACAA2 high | ACAA2 low |
|---|---|---|---|---|
| N | | 142 | 75 | 66 |
| Sex (%) | Female | 69 (48.6) | 31 (50.8) | 38 (46.9) |
| | Male | 73 (51.4) | 30 (49.2) | 43 (53.1) |
| Age (%) | >=65 | 65 (45.8) | 24 (39.3) | 41 (50.6) |
| | <65 | 77 (54.2) | 37 (60.7) | 40 (49.4) |
| Location (%) | Left | 70 (49.3) | 30 (49.2) | 40 (49.4) |
| | Right | 71 (50.0) | 31 (50.8) | 40 (49.4) |
| | NA | 1 (0.7) | 0 (0.0) | 1 (1.2) |
| TNM stage (%) | I | 12 (8.5) | 6 (9.8) | 6 (7.4) |
| | II | 59 (41.5) | 23 (37.7) | 36 (44.4) |
| | III | 41 (28.9) | 15 (24.6) | 26 (32.1) |
| | IV | 30 (21.1) | 17 (27.9) | 13 (16.0) |

N/n, number; T, tumor; N, Lymphnodes; M, metastasis; MMR, mismatch repair.
